# Public speaking training in front of a supportive audience in Virtual Reality improves performance in real-life

**DOI:** 10.1038/s41598-023-41155-9

**Published:** 2023-08-26

**Authors:** Leon O. H. Kroczek, Andreas Mühlberger

**Affiliations:** Department of Psychology, Clinical Psychology and Psychotherapy, University of Regensburg, Universitätsstraße 31, 93053 Regensburg, Germany

**Keywords:** Human behaviour, Psychology and behaviour

## Abstract

Public speaking is a challenging task that requires practice. Virtual Reality allows to present realistic public speaking scenarios in this regard, however, the role of the virtual audience during practice remains unknown. In the present study, 73 participants completed a Virtual Reality practice session while audience was manipulated to be supportive or unsupportive or presentations were practiced without audience. Importantly, following the virtual practice, participants held the presentation during a real university course via Zoom. We measured emotional experience, self-efficacy, and the subjective evaluation of performance at baseline, after VR practice, and after the real presentation. Additionally, participants’ performance in the real presentation was evaluated by instructors (blinded to condition). Supportive in contrast to unsupportive audiences led to more positive believes about one’s own performance, while there were no changes in beliefs in the group without audience. Importantly, practice in front of a supportive compared to unsupportive audience resulted in a more positive evaluation of speaker confidence in real-life public speaking as rated by the instructors. These results demonstrate an impact of virtual social feedback during public speaking on subsequent subjective performance evaluation. This may increase self-confidence resulting in actual improved public speaking performance in real-life.

## Introduction

Public speaking is a frequent yet challenging task in many professions. Everyone who has ever stood in front of an audience knows that it can be hard to perform. Although some people may find it easier than others to give structured, informative, and engaging presentations, most people require extensive practice to improve their presentation skills^1^. While schools and academic programs typically include public speaking as part of their curricula the amount of practice opportunities is limited. Furthermore, previous research has shown that practice situations need to be close to the test situation in order to be effective in improving performance^2^. Practicing in front of an audience, however, may be even more difficult to realize for most people than practicing alone. Virtual Reality (VR) has been suggested as a potential solution to this problem, allowing one to practice public speaking in a realistic scenario in front of a virtual audience. Previous studies found that public speaking in front of a virtual audience elicits similar experiences and physiological reactions as public speaking in front of a real audience^3^. Furthermore, speech rehearsal in Virtual Reality was positively evaluated by students and was found to improve voice quality compared to Non-VR trainings^4,5^. Overall, these studies suggest that VR can be used to increase performance in public speaking, however, it remains unknown under which circumstances VR practice can be most efficient in increasing performance in real-life tests.

Social anxiety disorder (SAD) is characterized by the fear of negative evaluation by others and a fear of public embarrassment or humiliation^6^. For people suffering from social anxiety disorder, public speaking is highly anxiety-evoking and is typically avoided. Fear of public speaking, however, is also common in non-clinical samples and has been correlated with decreased public speaking performance^2^. Exposure to public speaking as component of cognitive behavioral therapy is an effective treatment of social anxiety and the fear of public speaking^7^. However, as mentioned above, opportunities for public speaking may be difficult to realize within a therapeutic session. Therefore, Virtual Reality exposure therapy has been suggested as a tool to present reproducible and controlled public speaking situations *in virtuo* (for a review see^8^). While several studies have demonstrated that VRET can be an effective treatment for SAD and the fear of public speaking^9,10^, there are still mixed results regarding the question whether exposure in virtuo can be as effective as exposure in vivo^11,12^*.* This has been explained by the challenge of creating realistic and immersive social scenarios in Virtual Reality. For instance, Owens and Beidel^13^ found that giving a speech in front of a virtual compared to a real audience elicited less physiological arousal, while another study found similar physiological reactions between real and virtual public speaking scenarios^3^. Differential reactions towards real-world and VR scenarios could also be linked to presence, i.e. the feeling of being in a virtual environment^14^, and more specifically social presence, i.e. the feeling of being in the virtual environment together with other persons^15^. A recent study demonstrated that social presence influenced the degree to which audience behavior affected participants’ emotional experience in a public speaking scenario^16^. Overall, VRET can be effective in the treatment of SAD fear of public speaking, but more studies are required to investigate the contributing mechanisms.

The audience is the most important component of every public speaking situation. Audience behavior, i.e. visual or auditory reactions of the audience, serve as a social feedback for the person giving the speech and can be seen as an evaluation of a speaker’s performance. The evaluative nature of audience behavior makes it also highly relevant with respect to the fear of public speaking. Previous studies have investigated which behaviors are linked to specific characteristics of a virtual audience. Using both screen-based as well as immersive paradigms, several nonverbal cues could be identified that are linked to particular audience attitudes^17–19^. These studies demonstrate that manipulating behavior influences perceived engagement and valence of a virtual audience. Another line of research has focused on the effects that such audience behaviors evoke in the speaker^20–22^. A VR study compared supportive, neutral, and unsupportive audience behavior and found that fear of public speaking was mostly influences by unsupportive (i.e. disinterested) audience behavior^20^. Other studies, however, which compared supportive and unsupportive audience behavior in real-life public speaking scenarios found similar physiological responses to both audience behaviors^21^ or even increased physiological reactivity to supportive behavior^23^. The latter finding was interpreted by the authors in terms of an increase in speakers’ effort that was triggered by the positive audience feedback. It remains unclear whether supportive audience behavior in Virtual Reality might be less effective in realizing the feeling of a positive social evaluation indicated by a supportive audience. On a perceptual level, however, speakers seem to be able to differentiate supportive and unsupportive audience behavior in VR^16,19^. Another mechanism mediating the relationship of audience behavior and public speaking might be self-efficacy^4^. In line with this notion, a recent study could show that participant’s belief about their performance in a public speaking task influenced quality of performance as rated by others^24^. Having the impression to give a good performance as indicated by positive feedback from the audience might therefore increase self-efficacy and actual performance. This is also supported by a recent study, where public speaking training with an interactive compared to a passive virtual audience was found to increase motivation and engagement, even though no direct effects of audience feedback were found on behavioral performance^22^. Overall, while characteristics of audience attitudes have been investigated in virtual scenarios^17–19^, it remains unclear whether such attitudes affect one’s own believes about performance as well as performance in real-word public speaking.

To answer this question, the present study manipulated audience behavior in a public speaking practice session conducted in Virtual Reality while emotional experience and the speaker’s own believes about performance were assessed. Crucially, we also assessed quality of performance in a subsequent real-world public speaking performance in a university seminar rated by the course instructors (blinded to the experimental condition). Audience behavior in the VR practice session was designed to be either *supportive* or *unsupportive*. In addition, a control group held the practice speech in front of an empty virtual room with *no audience*. This allowed to investigate whether audience behavior in a VR public speaking training influences emotional experience, subjective beliefs about performance, and influences performance in the real world. We expected that *supportive* compared to *unsupportive* audience behavior would result in lower fear as well as more positive belief about one’s own performance. In addition, we expected that supportive compared to unsupportive audience behavior would result in a more positive belief about one’ own performance and a better public speaking performance in the real world.

## Results

### Emotional experience

Data analyses were conducted to test whether participant’s emotional experience (measured via ratings) was affected by public speaking in front of a virtual (*post VR-practice*) or real audience (*post Seminar*) compared to a baseline measurement before the virtual public speaking task. In addition, we investigated whether experiences would differ as a function of audience behavior.

#### Arousal

**Figure 1:**
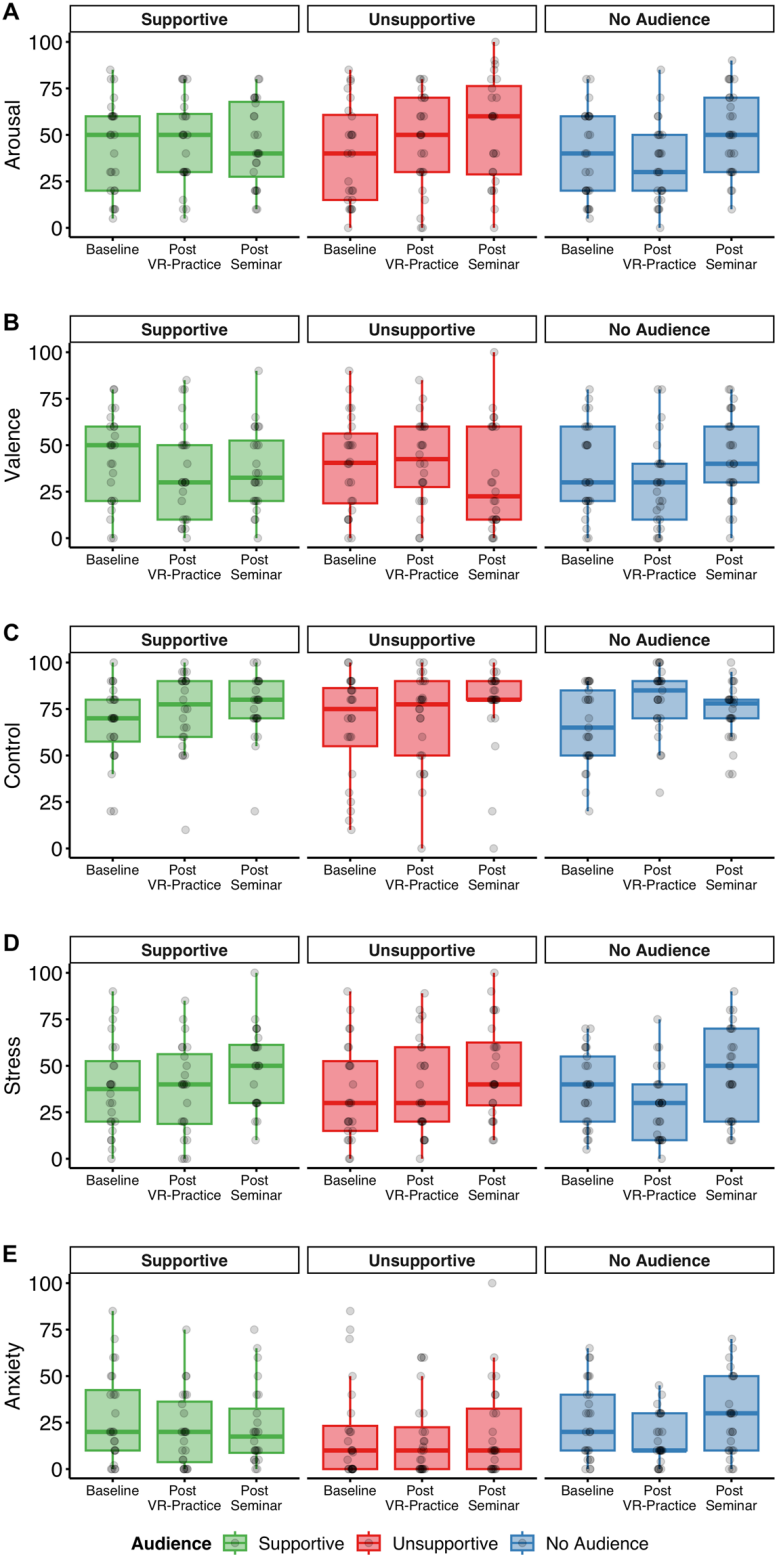
Ratings of (**A**) arousal, (**B**) valence, (**C**) feeling of control, (**D**) stress, and (**E**) anxiety as a function of time point (within-subject) and audience (between-subject). See Supplementary Material Fig. S1 for an illustration where different audience behaviors are aggregated within time points.

A mixed ANOVA with the factors Audience and Time Point revealed only a main effect of Time Point, F(2,140) = 4.75, p = 0.010, η_p_^2^ = 0.06. There was no significant main effect of Audience, F(2, 70) = 0.31, p = 0.735, η_p_^2^ < 0.01, and no interaction between Audience and Time Point, F(4, 140) = 1.64, p = 0.168, η_p_^2^ = 0.04. Post-hoc t-tests showed that arousal *post Seminar* was rated significantly higher than arousal *post VR-practice*, t(72) = 2.61, p = 0.033, d = 0.31, and arousal at *Baseline*, t(72) = 2.47, p = 0.033, d = 0.29. Arousal ratings *post VR-practice* did not differ significantly from arousal ratings at *Baseline*, t(72) = 0.36, p = 0.722, d = 0.04. Overall, arousal was higher during the real presentation than during the VR presentation but did not differ between audience groups (Fig. 1A).

#### Valence

The mixed ANOVA showed no main effect of Audience, F(2, 70) = 0.07, p = 0.936, η_p_^2^ < 0.01, no main effect of Time Point, F(2,140) = 0.70, p = 0.499, η_p_^2^ < 0.01, and no interaction effect, F(4, 140) = 1.96, p = 0.105, η_p_^2^ = 0.05. Unpleasantness of public speaking did not differ with respect to audience group and did not differ between presenting in the VR practice or the real-life seminar (Fig. 1B).

#### Control

Analysis of participants’ feeling of control for different audience groups and time points showed a main effect of Time Point, F(2,140) = 5.77, p = 0.004, η_p_^2^ = 0.08, but no effect of Audience, F(2,70) = 0.13, p = 0.879, η_p_^2^ = 0.08, and no interaction effect, F(4, 140) = 1.03, p = 0.396, η_p_^2^ = 0.03. Post-hoc t-tests showed that feeling of control significantly increased from *Baseline* to *post VR-Practice*, t(72) = 2.57, p = 0.027, d = 0.30, and from *Baseline* to *post Seminar*, t(72) = 3.07, p = 0.009, d = 0.36. There was no significant change in the feeling of control between *post VR-Practice* and *post Seminar* ratings, t(72) = 0.46, p = 0.647, d = 0.05 (Fig. 1C).

#### Stress

The mixed ANOVA on stress ratings revealed a main effect of Time Point, F(2, 140) = 10.58, p < 0.001, η_p_^2^ = 0.13, but no main effect for Audience, F(2, 70) = 0.26, p = 0.771, η_p_^2^ < 0.01, and no interaction effect, F(4, 140) = 0.64, p = 0.632, η_p_^2^ = 0.02. Post-hoc tests showed that stress ratings were significantly increased *post Seminar* compared to *Baseline*, t(72) = 3.98, p < 0.001, d = 0.43, and *post VR-Practice*, t(72) = 3.70, p < 0.001, d = 0.47. There was no change in stress ratings between *Baseline* and *post VR-Practice* assessment, t(72) = − 0.77, p = 0.440, d = 0.09. Overall, stress ratings were higher for the seminar presentation compared to the practice presentation but did not differ by audience group (Fig. 1D).

#### Anxiety rating

The analysis of anxiety ratings revealed a main effect of Time Point, F(2, 140) = 3.79, p = 0.025, η_p_^2^ = 0.05, but no effect of Audience, F(2, 70) = 0.58, p = 0.562, η_p_^2^ = 0.02, and no interaction effect, F(4, 140) = 0.90, p = 0.466, η_p_^2^ = 0.03. Post-hoc t-tests showed that anxiety ratings were significantly lower *post VR-Practice* compared to *Baseline*, t(72) = 2.56, p = 0.038, d = 0.30. Higher anxiety rating were reported *post Seminar* compared to *post VR-Practice*, t(72) = 2.38, p = 0.040, d = 0.28. There was no difference in anxiety ratings between *Baseline* and *post Seminar*, t(72) = 0.18, p = 0.857, d = 0.02. In sum, participants’ feeling of anxiety was reduced by the VR practice session but then increased for the seminar presentation (Fig. 1E).

### Fear of public speaking—PRCS

Participants’ general fear of public speaking was assessed at baseline and after the presentation in the seminar using the *Personal Report of Confidence as a Speaker* questionnaire (PRCS)^25^. A mixed ANOVA revealed a marginal significant effect of Time Point, F(1,68) = 3.87, p = 0.053, η_p_^2^ = 0.05, but no effect of Audience*,* F(1,68) = 0.03, p = 0.967, η_p_^2^ < 0.01, and no interaction effect, F(2, 68) = 0.94, p = 0.395, η_p_ = 0.03. Fear of public speaking decreased over the course of the study over all audience groups (*Baseline*: Mean = 11.09, SD = 6.21; *post Seminar*: Mean = 10.41, SD = 6.89).

### Self-evaluation of performance

Participants evaluation of their own presentation performance was measured as general self-efficacy via GSE questionnaire as well as participants’ rating of their believe about the own performance.

#### Self-efficacy

**Figure 2:**
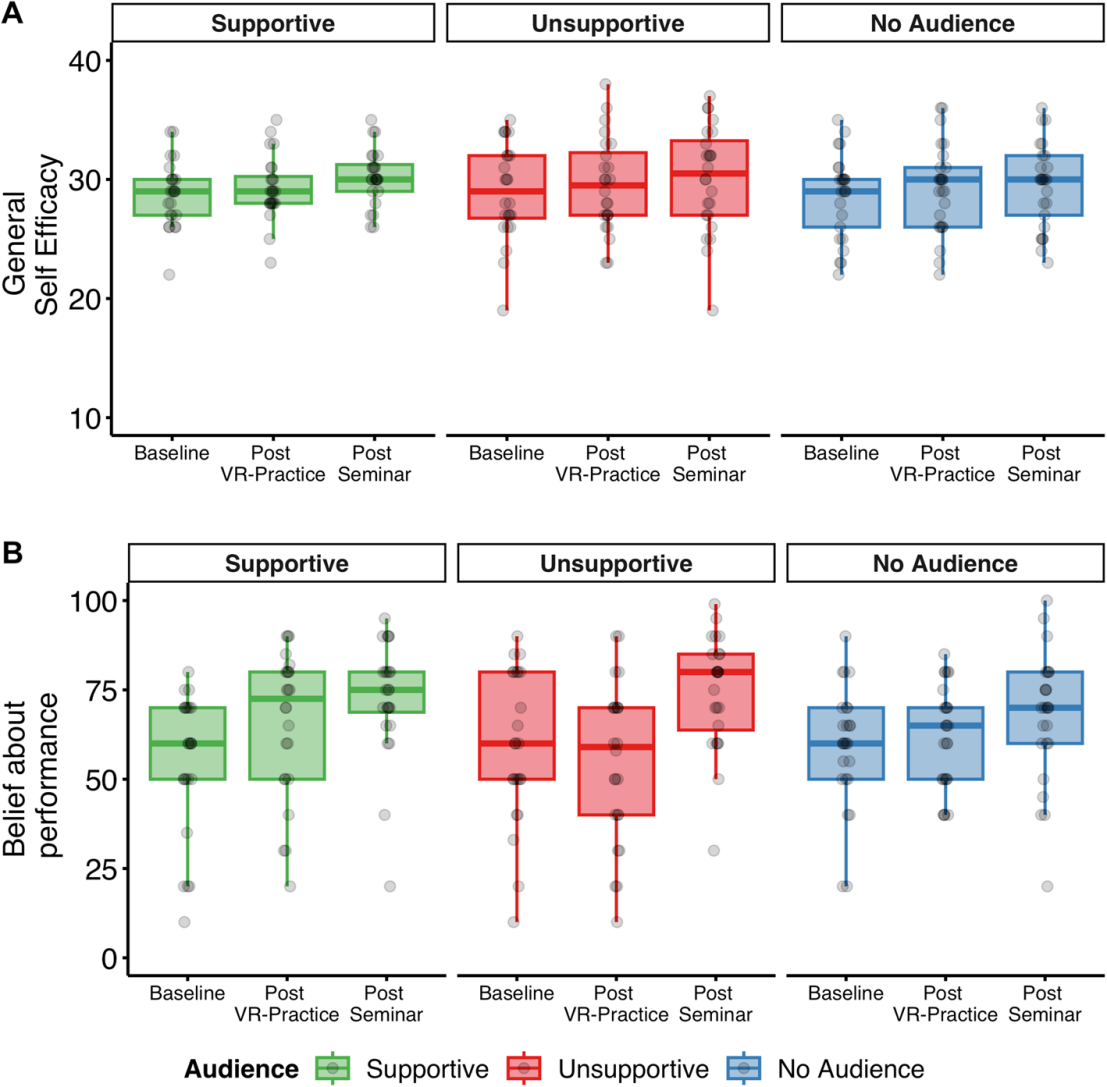
(**A**) Self efficacy as assessed via the GSE and (**B**) subjective belief about one’s performance. Individual data points shown as a function of Time Point (within-subject) and Audience (between-subject). See Supplementary Material Fig. S2 for an illustration where different audience behaviors are aggregated within time points.

General self-efficacy measured via the GSE questionnaire was analyzed with respect to Time Point and Audience Behavior (Fig. 2A). The analysis revealed a main effect of Time Point, F(1.56, 109.29) = 6.93, p = 0.003, η_p_^2^ = 0.09 (ε = 0.78), but no effect of Audience, F(2, 70) = 0.10, p = 0.904, η_p_^2^ < 0.01, and no interaction effect, F(3.12, 109.29) = 0.24, p = 0.877, η_p_^2^ < 0.01 (ε = 0.78). Post-hoc t-tests revealed that self efficacy increased from *Baseline* to *post VR-Practice*, t(72) = 2.52, p = 0.028, d = 0.30, and from *Baseline* to *post Seminar*, t(72) = 3.10, p = 0.008, d = 0.36. There was no significant increase from *post VR-Practice* to *post Seminar*, (t(72) = 1.91, p = 0.060, d = 0.22. In sum, the general measure of self-efficacy was increased after the VR practice and this increase was maintained after the seminar presentation.

#### Subjective belief about performance

As a subjective measure of public speaking performance, participants rated their believes about their presentation performance (Fig. 2B). A mixed-effect ANOVA revealed an interaction effect between Time Point and Audience, F(3.36, 117.46) = 2.77, p = 0.039, η_p_^2^ = 0.07 (ε = 0.84), a main effect of Time Point, F(1.68, 117.46) = 17.88, p < 0.001, η_p_^2^ = 0.20 (ε = 0.84), but no main effect of Audience, F(2, 70) = 0.07, p = 0.928, η_p_^2^ < 0.01.

Post-hoc t-test were conducted to follow-up on the interaction effect. When audience showed supportive behavior there was a more positive belief about performance from *baseline* (M = 54,38, SD = 19.91) to the assessment *post Seminar* (M = 72.50, SD = 16.55), t(23) = 3.35, p = 0.008, d = 0.68. Believes about performance *post VR-practice* were at an intermediate level (M = 65.71, SD = 20.46), with only marginal significant differences to either *baseline*, t(23) = 2.24, p = 0.070, d = 0.46, or the assessment *post Seminar*, t(23) = − 2.24, p = 0.070, d = -0.46.

In contrast, when comparing believes about performance in the group with unsupportive audience behavior, we found that believes about performance did not change from *baseline* (M = 59.08, SD = 21.35) to *post VR-practice* (M = 54.92, SD = 22.44), t(23) = − 1.12, p = 0.273, d = − 0.22. But there was a significant increase in believes about performance *post Seminar* (M = 74.54, SD = 15.87) compared to assessment *post VR-practice*, t(23) = 5.41, p < 0.001, d = 1.11, or at *baseline*, t(23) = 3.93, p = 0.001, d = 0.80. There were no changes in believes about performance between *baseline*, *post VR-practice*, and *post Seminar* in the no audience group (Baseline: M = 59.20, SD = 16.87; Post VR-Practice: M = 61.80, SD = 14.35, Post Seminar: M = 67.40, SD = 18.14; all comparisons p > 0.05).

Finally, we compared whether groups differed in changes from baseline either at post VR-practice or post Seminar. There was a marginal significant difference in changes about believes from *baseline* to *post VR-practice* between the group with supportive audience behavior (M_Diff_ = 11.33, SD_Diff_ = 24.77) compared to the group with unsupportive audience behavior (M_Diff_ = -4.17, SD_Diff_ = 18.16), t(42.18) = 2.36, p = 0.053, d = 0.71. There were no other group differences in changes about believes from baseline (all p > 0.10).

In summary, these results demonstrate that audience group influenced believes about performance in the practice session but not in the seminar. Furthermore, believes about performance when giving the actual presentation were higher than at practice or baseline.

### Presence

Social and physical presence during the VR practice was assessed with the IPQ and MPS questionnaires and analyzed using ANOVA with the between-subject factor Audience Behavior (full results are presented in Table 1). There was a significant effect of Audience on Social Presence subscale of the MPS. Post-hoc t-tests revealed that social presence was increased for supportive audience behavior group compared to the no audience group, t(45.21) = 3.02, p = 0.012, d = 0.86. There was no difference between supportive and unsupportive audience group, t(45.11) = 1.20, p = 0.237, d = 0.35, and between unsupportive audience behavior group and no audience group, t(46.83) = 1.95, p = 0.115, d = 0.56. Public speaking in front of a supportive audience was related to the highest rating of social presence.

**Table 1:** Group mean values and standard deviations (in brackets) for the general (G), spatial presence (SP), experience realism (ER), and involvement (INV) subscales of the iGroup Presence Questionnaire and the Physical and Social Presence subscale of the Multimodal Presence Scale.

Presence parameter	Audience group					
	Supportive audience	Unsupport. audience	No audience	F (2,70)	p	η_p_^2^
IPQ- G	4.50 (0.98)	4.45 (0.83)	4.24 (1.27)	0.44	0.646	0.01
IPQ-SP	4.59 (0.89)	4.72 (0.82)	4.51 (0.75)	0.38	0.683	0.01
IPQ-ER	3.06 (0.84)	2.94 (0.91)	2.91 (0.81)	0.22	0.804	< 0.01
IPQ-INV	4.20 (1.20)	4.12 (1.31)	3.50 (1.24)	2.31	0.106	0.06
MPS physical	3.68 (0.77)	3.81 (0.60)	3.79 (0.59)	0.26	0.773	< 0.01
MPS social	3.06 (0.89)	2.77 (0.78)	2.34 (0.76)	4.86	0.011	0.12

### Other-evaluation of performance

**Figure 3:**
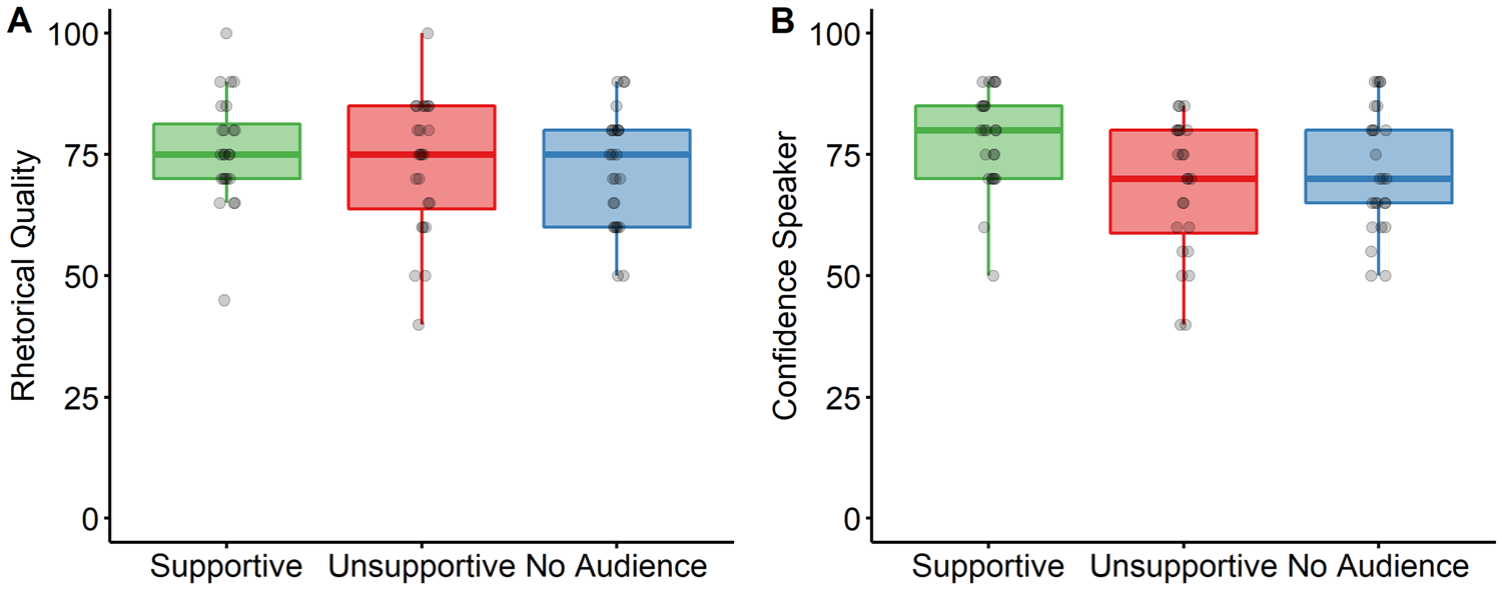
Course instructors’ evaluation of public speaking performance in the seminar with respect to rhetorical quality (**A**) and confidence (**B**) as a function of audience group.

Finally, we also measured participants’ performance during their public speaking presentations in front of a real audience in an online seminar**.** Course instructors (blinded to the experimental manipulation) were asked to rate rhetorical quality as well as speaker confidence of participants’ presentations in the seminar (Fig. 3). While, there was no effect of Audience on the ratings of rhetorical quality, F(2,70) = 0.76, p = 0.470, η_p_^2^ = 0.02, there was a significant effect of Audience on ratings of speaker confidence, F(2,70) = 4.18, p = 0.019, η_p_^2^ = 0.11. Post-hoc t-tests revealed that speakers in the supportive audience group were rated as significantly more confident than speakers in the unsupportive audience group, t(42.19) = 2.90, p = 0.018, d = 0.84. There was no significant difference between the supportive audience group and the group with no audience, t(45.64) = 1.93, p = 0.120, d = 0.55, or between the unsupportive group and the group with no audience, t(46.16) = − 1.02, p = 0.312.

## Discussion

Participants completed a Virtual Reality public speaking practice followed by a performance in real life. During the VR practice session audience behavior was manipulated to be either supportive, unsupportive or no audience was presented. Interestingly, performance in real-life was affected by behavior of the virtual audience during the practice session. We found that audience behavior influenced both believes about one’s own performance in the VR practice session as well as the evaluation of confidence in real-life public speaking though blinded course instructors. The most beneficial effects were found for supportive audience behavior. These results add to the existing evidence on efficacy of a Virtual Reality on public speaking performance^4,5^ and highlight the role of social feedback and audience behavior in virtual settings.

Overall, our data suggest that public speaking in front of a supportive audience increased positive beliefs about ones’ performance. Furthermore, a positive belief about one’s performance resulted in increased speaker confidence during real-life public speaking. This is in line with recent findings that demonstrate a link between beliefs about performance and the actual evaluation of one’s performance^24^. Importantly, the present results extend these findings, by demonstrating that also the beliefs about past performance can influence upcoming public speaking performance and be transferred to real-world public speaking situation. Moreover, Virtual Reality settings with virtual audiences can modulate these beliefs. These results have implications about the use of Virtual Reality in educational settings and highlight supportive audience behavior as a crucial factor to increase performance. Increased social presence that was related to supportive audiences might contribute to this effect by rendering virtual public speaking scenarios to be more similar to real-life situations^15^. This can be effective as the similarity between practice and test situations has been suggested as a predictor of practice success^2^. Overall, Virtual Reality scenarios including supportive audience behavior seems to be a promising tool to enhance public speaking performance.

Besides changes related to the evaluation of performance, we also obtained measures regarding emotional experience of public speaking in VR practice and the seminar. While we observed overall changes in these domains across the course of the study, there was no modulation of effects by audience behavior. Given that the VR practice session and the real-life presentation in the seminar differed with respect to several aspects, it is important to differentiate between effects that can be related to VR practice and effects that can be explained by task demands or contextual factors (for instance, presentations in the seminars were longer than the practice talks and included questions from the audience and the course instructor). A VR practice effect, i.e. change from baseline to post VR-practice session that was maintained in the post Seminar assessment, was found for the feeling of control. This suggests that VR practice improved participants control of a public speaking situation. This was also reflected in the general self-efficacy questionnaire, for which the same pattern was observed. In contrast, we found that both arousal and stress ratings were not affected by the VR practice but increased when comparing public speaking in the seminar compared to VR practice. In line with previous findings^13^, these data suggest that real-life public speaking was more stressful and arousing than public speaking in VR. However, it should be noted that there were several differences between the VR-practice and real-life seminar situation that can account for this effect. For instance, public speaking in the seminar was conducted in front of a course instructor who evaluated the content and performance of participants’ presentations, but there was no such evaluation in the VR practice. Furthermore, only the real-life presentation included questions by either course instructor or seminar attendees and the real-life presentations were longer (duration around 1 h) than the 10 min VR practice. Interestingly, the feeling of anxiety showed both a decrease from baseline to post VR practice and an increase from post VR practice to post seminar. This finding suggests that the public speaking in VR reduced anxiety but that the real-life compared to VR situation was more anxiety provoking after all. In addition, we observed a decrease of fear of public speaking over the course of the study supporting previous findings where exposure to public speaking resulted in reduced fear^9,10,26,27^. However, the current study did not include a non-practice or non-exposure condition, therefore changes in fear of public speaking could be driven by other factors. This effect should therefore be examined in further randomized-controlled trials.

In the present study we did not find an effect on audience behavior on the feeling of pleasantness during the VR practice. This finding was unexpected as a previous study found that supportive and unsupportive audience behavior differed with respect to valence^16^. One possible explanation for this discrepancy might be related to the presentation mode. In the present study, participants could see their actual presentation slides on a computer screen during the VR presentation, while in Pfaller et al.^16^ only the topic was presented on the screen. As a result, participants might have been less attentive to the audience, especially, when negative audience behavior was shown, resulting in diminished differences between conditions. Furthermore, while Pfaller et al., implemented audience behavior as a within-subject factor, audience behavior was manipulated as a between subject factor in the present study. Consequently, effects might have been too small to detect with the present sample.

To our knowledge the present study is the first to manipulate audience behavior in a Virtual Reality practice session and measure effects in real-life public speaking, however, there are some limitations that should be discussed. First, the real-life public speaking performance was conducted only via a video communication platform (Zoom), leading to a reduced public speaking situation compared to a setting where presenter and audience are in the same room. This might have affected the experience and the performance of participants and thus reduces generalizability and external validity of the present results. Future studies should therefore test Virtual Reality practice on performance in in-person situations. However, it should be noted that regardless of the digital format, the presentation in the seminars were experienced as more stressful and arousing than the practice session. One could speculate that practice effects might be even more prominent when public speaking in front of an audience is more challenging. Another limitation of video-based seminars is that the course instructors had only restricted access to the presenter behavior (face and voice). As a result, course instructors might have focused more on verbal parameters and less on non-verbal aspects, such as gestures or body posture to evaluate speaker confidence. While, face-to-face situations might provide enriched information, it should be noted that a previous study found a specific improvement in vocal parameters by public speaking training and that this aspect was preserved in the digital format of the seminars^5^.

Another point that should be discussed is audience engagement. In the present study the unsupportive audience was programmed to show disinterested behavior. Disinterest behaviors, like averted gaze, have been shown to result in low evaluations of audience arousal and might therefore account for the present finding of reduced social presence in the unsupportive group^17,18^. It is important to note, that low engagement of an audience might in fact lead to high arousal in the person giving the presentation, as low engagement might be interpreted as a negative evaluation of one’s performance^16^. While the goal of the present study was to investigate general effects of audience behavior on performance in real-life situations, future studies should investigate the role of specific behavioral patterns.

Finally, it should be acknowledged that the present study was conducted with an unselected student sample, not a clinical sample with a high fear of public speaking. Our healthy participants might have experienced the situation as generally less threatening and unpleasant compared to persons suffering from social anxiety disorder. This might have implications on affective reactions to the different audience behaviors. For instance, Slater et al.^28^ found that public speaking in Virtual Reality only increased anxiety in persons with public speaking phobia. Future studies should therefore investigate audience behavior in high social anxious individuals.

In conclusion, the current study demonstrates that supportive audience behavior in a Virtual Reality public speaking practice can enhance positive beliefs about one’s performance and results in a more positive evaluation of performance by others in a real-life public speaking situation. This highlights the role of virtual audiences and has direct implications on the design of VR practice for the purpose of improving public speaking performance.

## Methods

### Participants

Overall 73 students of the Bachelor of Science (undergrad) Psychology program at Regensburg University were recruited. All students participated in one of three seminars covering “Clinical Psychology and Neuropsychology”. Sample size was not determined before the start of the experiment but was determined as the maximum number of students within the seminars who were willing to participate in the study. A sensitivity analysis was conducted using MorePower (v 6.0.4)^29^ for a mixed 3 by 3 ANOVA with a power of 1-beta = 0.80, alpha = 0.05, and sample size of N = 73. The analysis revealed that the sample size allowed to detect a *medium-to-big* effect size of f = 0.379 (η_p_^2^ = 0.13) for between-subject effects, a *medium* effect size of f = 0.265 (η_p_^2^ = 0.07) for within-subject effect, and a *medium* effect of f = 0.297 (η_p_^2^ = 0.08) for an interaction effect.

Participants were randomly assigned to one of the three experimental groups: “Supportive Audience” (n = 24), “Unsupportive Audience” (n = 24), “No Audience” (n = 25). Participants did not report any neurological or mental disorders. Participants received a compensation of 15 € or course credits for participation. Demographic data of the groups are summarized in Table 2. There was only a significant difference between groups on age. This effect was found to be driven by single datapoint. The study was in accordance with the Declaration of Helsinki. Study procedures were reviewed and approved by the Regensburg University ethics committee and all participants gave their informed consent in writing. The study was carried out according to approved procedures.

**Table 2:** Demographic data in the experimental groups.

Parameter	Audience group			Test statistic	p-value
	Supportive (n = 24)	Unsupportive (n = 24)	No audience (n = 25)		
Age	21.67 (1.90)	21.71 (1.68)	23.32 (3.74)	F(2,70) = 3.17	0.048
Sex	19 f/5 m	22 f/2 m	19 f/6 m	X^2^(2) = 2.28	0.320
Seminar membership	Seminar 1: 11	Seminar 1: 6	Seminar 1: 7	X^2^(4) = 3.95	0.413
	Seminar 2: 12	Seminar 2: 4	Seminar 2: 8		
	Seminar 3: 7	Seminar 3: 5	Seminar 3: 13		
SPIN	16.87 (6.37)	20.17 (10.27)	18.32 (10.53)	F(2,70) = 0.76	0.472
BFNE	38.50 (7.49)	43.29 (8.91)	40.80 (8.39)	F(2,70) = 2.00	0.142

For age, SPIN scores, and BFNE scores group mean values and standard deviations (in brackets) are displayed. Differences were tested using one-factorial ANOVAs and test statistics and p-values are reported. For Sex and Seminar, we report number of participants in category per group. Here, Chi-square test were used to test whether numbers differed between groups.

### Experimental design

Behavior of the virtual audience was manipulated as a between-subject factor with three factor levels (*Supportive audience*, *Unsupportive audience*, *no audience*). Participants’ reports regarding emotional experience and performance were measured at three different timepoints throughout the study: (1) at *Baseline* before the VR, (2) after the *VR practice,* and (3) after the real-life presentation in the *Seminar* (*Time Point* as 3-level within-subject factor: *Baseline*, *post VR-practice*, *post Seminar*). Additionally, fear of public speaking was measured via questionnaires at *Baseline* and *post Seminar* (*Time* Point as 2-level within-subject factor). Finally, performance evaluation through the course instructors was obtained after the presentation in the *Seminar*.

### Apparatus

#### VR Set-Up

Virtual Reality was presented via Head Mounted Display (HMD, Vive Pro Eye, HTC, Taoyuan City, Taiwan) and rendered via the Unreal Game Engine (v 4.25.1, Epic Games Inc., Raleigh, USA) with experiment control plugin (VrSessionModUDK 1.0.16, VTplus GmbH, Würzburg, Germany). Virtual environments and virtual audience were controlled using TypeScript based control scripts (ExpoControl 1.5.23, VTplus, Würzburg, Germany). Participants uploaded their own presentation slides to be displayed in Virtual Reality. Navigating through slides was achieved via the left and right buttons on the VR motion controllers, visualized as a virtual presenter. The virtual environment consisted of two rooms: A lobby where participants could prepare their upcoming presentation on a virtual laptop as well as a lecture hall where participants were placed in front of eight tables. In the lecture hall a computer screen was placed front of the participants where they could see their slides.

**Figure 4:**
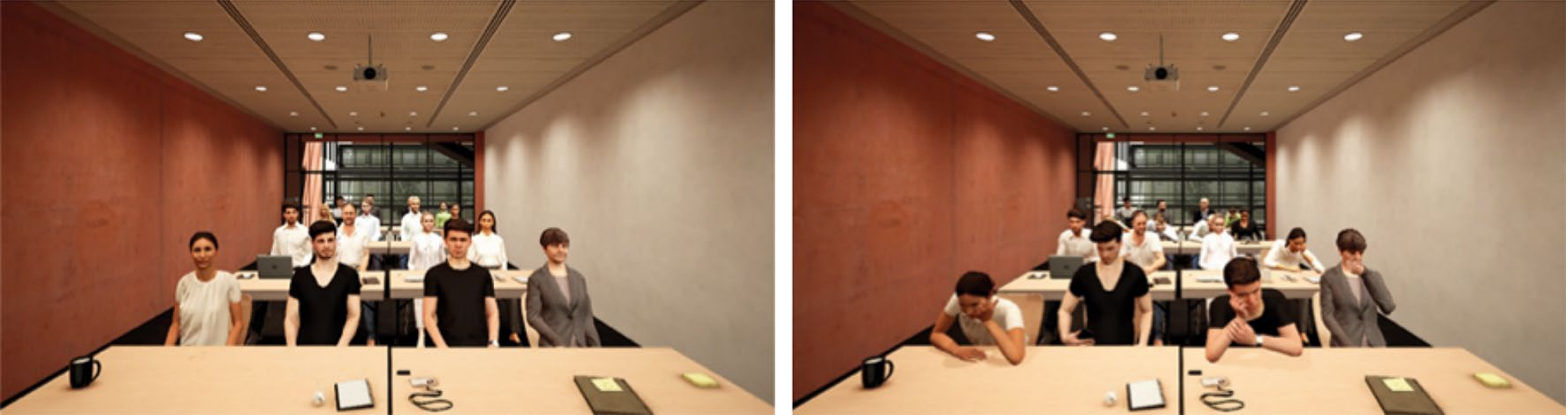
Supportive (left) and unsupportive (right) virtual audience behavior. The same scene without agents was presented in the no audience condition.

In the supportive and unsupportive audience condition, 16 virtual agents (8 male, 8 female) were placed at the tables facing the participant. No virtual agents were sitting at the tables in the no audience condition. Two distinct audience behaviors were implemented to differentiate between supportive and unsupportive audiences (Fig. 4 shows example scenes of the virtual environment including virtual audience, note that the persons shown do not depict real persons). Supportive behavior was operationalized as smiling facial expression, an open body posture, gaze directed towards the participant (always), and occasional nodding. Unsupportive behavior was operationalized as a disinterested audience with bored expressions, occasional yawning, posture leaning backwards or with arms crossed on the table and agents’ gaze directed away from the participant (always), either steering in the air or looking at a smartphone or laptop (gaze targets changed in random order every 3–10 s). Similar behaviors have been reported in previous studies that aim at characterizing audience attitudes from non-verbal cues^17–19^. Audience behaviors were shown by all virtual agents throughout the scenario. Animations of nonverbal behaviors appeared in random order with a probability of 0.95 for idle behaviors and a probability of 0.05 for characteristic behaviors (e.g. nodding for supportive or yawning for unsupportive audiences). There was no mix of supportive or unsupportive audience behaviors among the audience. According to Valence-Arousal models^19^, these different behavior should result in two distinct characterization of audience attitudes that differ in perceived valence and engagement. Importantly, the present audience behaviors have been evaluated in a previous study and were found to differ in valence, with supportive behavior being rated as more pleasant than unsupportive behavior^16^. The same study found no difference between supportive and unsupportive audience behaviors in the feeling of arousal during public speaking, but both audience behaviors lead to increased arousal compared to neutral audience behavior. Overall, these findings indicate that the two audience behaviors used in the present study can be used to provide speakers with different evaluations of their performance.

#### Questionnaires and ratings

All questionnaires were presented in German. Social phobia symptoms were assessed using the social phobia inventory (SPIN)^30,31^, as well as the Brief Fear of Negative Evaluation Scale (BFNE)^32^ and the *Personal Report of Confidence as a Speaker* questionnaire (PRCS)^25^. Self-efficacy was measured using the generalized Self Efficacy Scale (GSE)^33^. Presence was measured using the IGroup Presence Questionnaire (IPQ)^14^ with the subscales General (G), Spatial Realism (SR), Involvement (INV) and Experienced Realism (EXP). In addition, separate measures for Physical and Social Presence were assessed using the respective subscales of the Multimodal Presence Scale (MPS)^34^.

Participants were also asked to rate their subjective experience at several time point throughout the experiment. Ratings were given on a scale from 0 to 100 and were obtained for *Arousal* (very low arousal to very high arousal), *Valence* (very pleasant to very unpleasant), *Feeling of Control* (no control to full control), *Stress* (very low to very high), *Anxiety* (very low to very high), Belief about *Performance* (very bad to very good).

After the presentation in the real course, course instructors were asked to rate the presentation performance of the participant. These ratings were given on a scale from 0 to 100 for *Rhetorical Quality* (very low to very high) and Speaker *Confidence* (very low to very high).

### Procedure

The study consisted of a VR practice session that was conducted at the laboratory and a Seminar session where participants gave an online presentation in front of a real audience as part of an university course. The practice session was scheduled to be no earlier than 7 days before the real presentation (mean = 3.05 days, SD = 1.71). Before the VR practice session, participants were asked to send their presentation slides to the experimenter. The slides had to be part of the presentation that participants would give in the upcoming seminar. The topics varied between participants but were all related to the field of Clinical Psychology (e.g. classification of anxiety disorder). The slides were then loaded into the virtual scenario and were visible on a computer screen (see below).

#### VR practice session

Participants were screened for Covid-19 symptoms and received written experimental instructions and gave written informed consent. At the beginning of the study participants filled in questionnaires regarding demographic information and social anxiety symptoms (SPIN, BFNE). In addition, *Baseline* values were acquired for Confidence in Public Speaking (PRCS), Self-efficacy (GSE), as well as Arousal, Valence, Feeling of Control, Stress, Anxiety, and belief about performance. Participants were instructed to answer the rating questions with respect to the upcoming presentation.

In a next step, participants put on a HMD and were placed in the virtual environment. First, a preparation phase of 2 min was started. In this preparation phase, participants could get accustomed to the Virtual Reality, and they were asked to click through their slides which were presented on a virtual laptop in front of them using the motion controller. These instructions were provided by a male virtual agent standing next to the participant. After 2 min, the virtual agent guided participants to the lecture hall. Participants used the motion controller to navigate.

Once they arrived at the lecture hall participants were teleported to a position in front of a virtual audience or empty tables (depending on the experimental condition) and the presentation phase started. During the presentation phase, slides were displayed on a computer screen placed to the right of the participants and participants could use the motion controller to forward the slides. The slides were also presented on the wall behind the participants (simulated as a video projection from a virtual projector). Pressing the “up button” on the motion controller, allowed participants to use the controller as a laser pointer. Participants were instructed to give a 10 min presentation as they would do in real life. As the full presentations were longer than 10 min, participants were asked to practice only the initial 10 min of their presentations. After 10 min participants were asked to stop their presentation and the VR practice ended. At the end of the session participants then gave another round of ratings (*post VR-Practice*) regarding emotional experience (Valence, Arousal, Control, Stress, Anxiety) and their belief about their own presentation performance. In addition, participants filled in questionnaires regarding self-efficacy (GSE) and presence (IPQ, MPS). The practice session had a total duration of approximately 1 h.

#### Presentation in the seminar

Within one week after the practice presentation, participants held the presentation in a seminar in front of a real audience as part of their undergrad curriculum. There were three different seminars each supervised by a different course instructor (1 female, 2 male). Due to the Covid-19 pandemic all seminars were held as live, online courses via video communication platform Zoom (Zoom Inc., San José, USA). All course instructors were blinded to the experimental conditions and had been instructed to rate the rhetorical quality and confidence of participants presentations. After their presentations had been completed, participants received a link that allowed them to access an online survey provided via EvaSys (evasys GmbH, Lüneburg, Germany). In the online survey (*post Seminar*), participants were asked a final time to rate their emotional experience and belief about performance, as well as to fill in questionnaires regarding self efficacy (GSE) and confidence in public speaking (PRCS).

### Data processing and statistics

Data analysis was conducted in the R environment^35^ with the packages *tidyverse*^36^, *ezANOVA*, and *rstatix* installed. For analyses that included more than one timepoint for the same measure, a mixed ANOVA was calculated with *Audience* as a between-subject variable and *Time Point* as within-subject factor. In case of sphericity violations, Greenhouse–Geisser correction was applied^37^. Post-hoc t-tests were calculated to follow-up on significant main effects or interactions and Holm corrected for multiple comparisons^38^. Alpha level was set to 5%. Descriptive statistics (means and standard deviations) of all ratings are summarized in the supplementary material (Table S1). All analysis scripts are publicly available (https://osf.io/754yx/).

### Supplementary Information

Supplementary Information.

## Data Availability

Anonymized data are publicly available in an online repository (https://osf.io/754yx/).
