# Supplementary material for: Public speaking training in front of a supportive audience in Virtual Reality improves performance in real-life
Source: Sci Rep. 2023 Aug 26;13:13968. doi: 10.1038/s41598-023-41155-9 (PMC10460391; doi:10.1038/s41598-023-41155-9)
Supplement: Supplementary file 1 — Supplementary Information. [file 41598_2023_41155_MOESM1_ESM.pdf]

Supplementary Material: Public speaking training in front of a supportive audience in Virtual Reality improves performance in real-life

Leon O.H. Kroczek*[1] & Andreas Mühlberger[1]

[1] Department of Psychology, Clinical Psychology and Psychotherapy, University of Regensburg, Regensburg, Germany

Table S1: Mean values and standard deviations (in brackets) for ratings of emotional experience (arousal, valence, feeling of control, stress, anxiety), belief about performance, as well as questionnaires on general self efficacy (GSE) and confidence as a speaker (PRCS).

		**Audience Group**		
**Variable**	**Test Position**	**Supportive Audience**	**Unsupport. Audience**	**No Audience**
**Arousal**	*Baseline*	43.96 (24.76)	39.46 (26.76)	39.00 (23.89)
	Post VR-Practice-	45.63 (23.79)	45.63 (25.80)	34.60 (21.26)
	Post Seminar	45.29 (22.27)	52.21 (28.59)	51.80 (22.40)
**Valence**	*Baseline*	42.91 (24.04)	39.83 (25.60)	37.00 (24.45)
	Post VR-Practice-	35.63 (26.01)	41.67 (22.87)	29.68 (23.53)
	Post Seminar	37.08 (21.76)	32.08 (28.17)	43.80 (23.51)
**Control**	*Baseline*	66.88 (20.74)	66.04 (27.93)	65.60 (21.18)
	Post VR-Practice-	73.96 (21.31)	69.21 (25.46)	79.80 (18.00)
	Post Seminar	76.04 (16.93)	77.29 (22.98)	73.72 (15.54)
**Stress**	*Baseline*	38.13 (24.71)	35.42 (26.00)	37.60 (20.32)
	Post VR-Practice-	36.88 (24.66)	38.58 (26.19)	29.12 (19.14)
	Post Seminar	49.58 (21.41)	47.92 (25.75)	47.20 (23.98)
**Anxiety**	*Baseline*	27.79 (24.53)	19.58 (25.89)	26.20 (20.98)
	Post VR-Practice-	21.04 (20.22)	16.75 (20.86)	15.96 (13.42)
	Post Seminar	22.88 (21.40)	20.83 (25.05)	28.20 (21.35)
**GSE**	*Baseline*	28.79 (2.75)	29.00 (4.04)	28.64 (3.51)
	Post VR-Practice-	29.29 (2.68)	29.54 (3.96)	29.28 (3.75)

	Post Seminar	30.20 (2.36)	30.08 (4.44)	29.52 (3.55)
**Belief about performance**	*Baseline*	54.38 (19.91)	59.08 (21.35)	59.20 (16.87)
	Post VR-Practice-	65.71 (20.46)	54.92 (22.44)	61.80 (14.35)
	Post Seminar	72.50 (16.55)	74.54 (15.87)	67.40 (18.14)
**PRCS**	*Baseline*	11.67 (6.83)	11.13 (5.55)	10.50 (6.37)
	Post Seminar	10.21 (7.13)	10.17 (6.80)	10.84 (7.02)

Figure S1: Emotional Experience

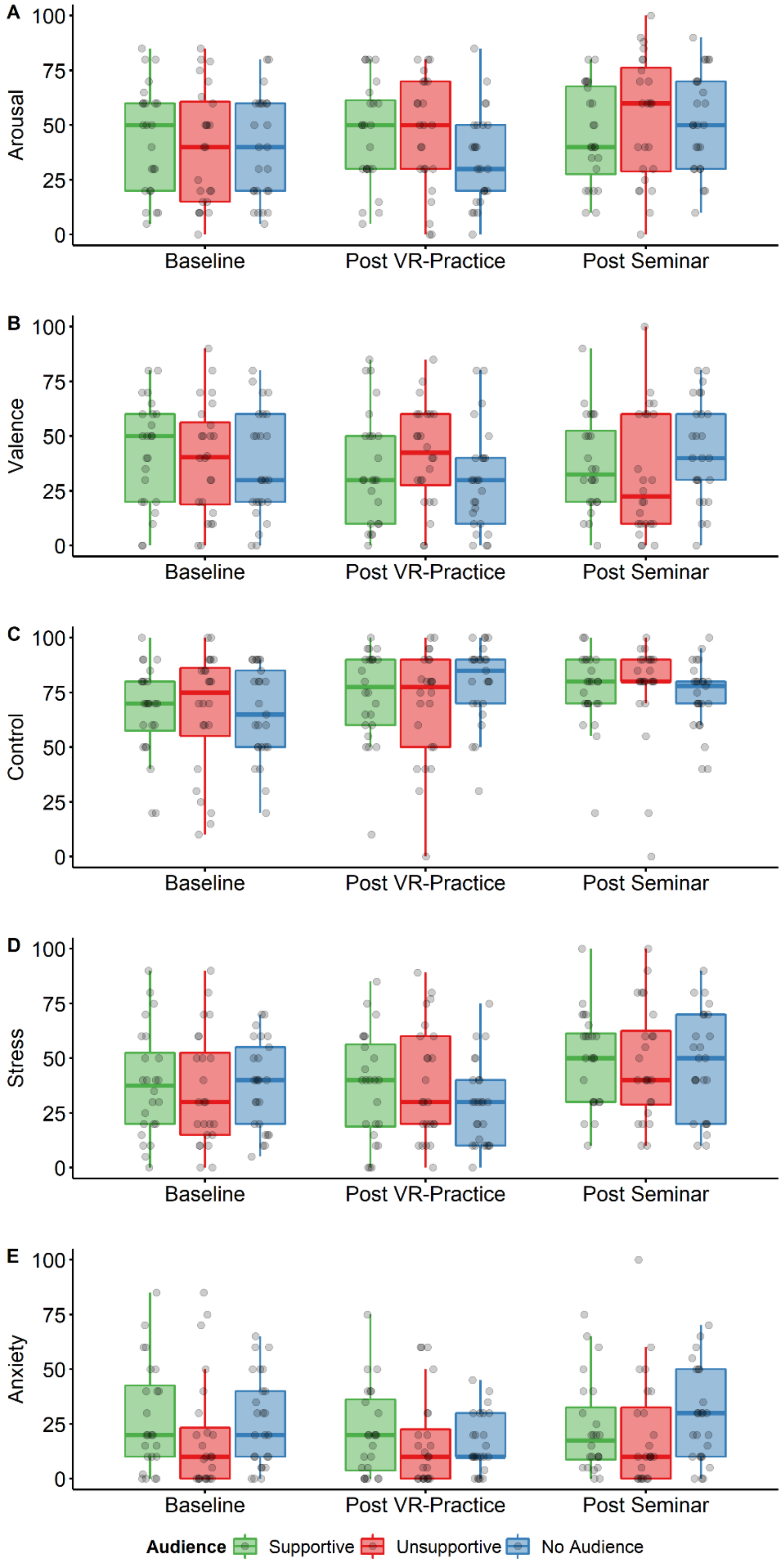

Figure S1: Ratings of (A) Arousal, (B) Valence, (C) Feeling of Control, (D) Stress, and (E) Anxiety as a function of Audience (between-subject) and Time Point (within-subject).

Figure S2: Self-evaluation of performance

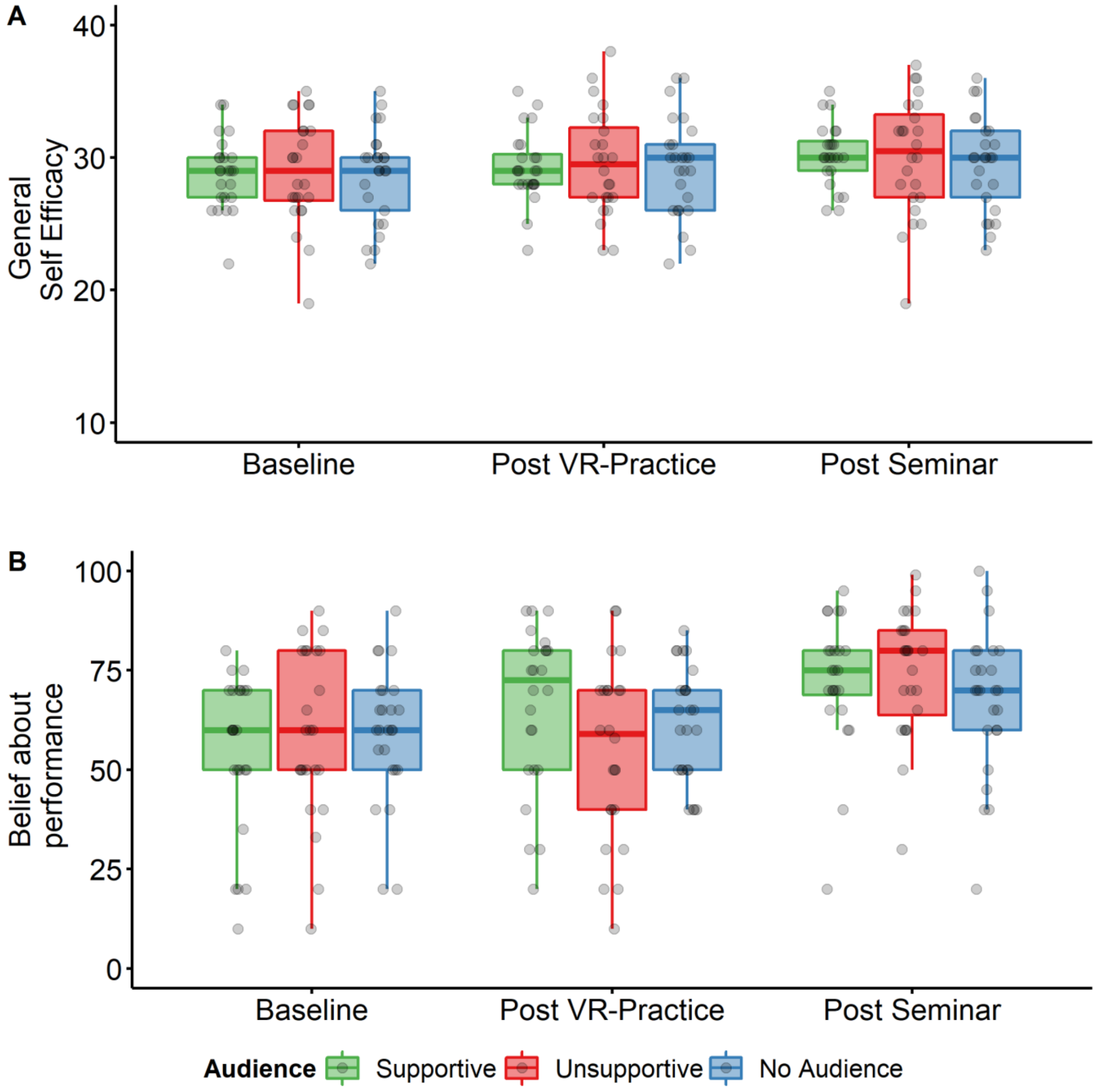

Figure S2: (A) Self efficacy as assessed via the GSE and (c) subjective belief about one's performance. Individual data points shown as a function of Audience (between-subject) and Time Point (within-subject).
